# Various levels of copra meal supplementation with *β*-Mannanase on growth performance, blood profile, nutrient digestibility, pork quality and economical analysis in growing-finishing pigs

**DOI:** 10.1186/s40781-017-0144-6

**Published:** 2017-07-14

**Authors:** H. J. Kim, S. O. Nam, J. H. Jeong, L. H. Fang, H. B. Yoo, S. H. Yoo, J. S. Hong, S. W. Son, S. H. Ha, Y. Y. Kim

**Affiliations:** 1 Department of Agricultural Biotechnology, College of Agriculture and Life Sciences, Seoul National University, 1 Gwanak-ro, Gwanak-gu, Seoul 08826 Republic of Korea; 2 PuKyung Pig Farmers Agricultural Cooperative, Gimhae, 50925 Republic of Korea

**Keywords:** Copra meal, *β*-mannanase, Growth performance, Economical analysis, Growing-finishing pigs

## Abstract

**Background:**

To reduce use of main feed ingredient like corn, soy bean meal (SBM) and wheat, alternative ingredients has been studied like copra meal (CM). Production amount of CM which has been high makes CM to be an alternative feed stuff. However, low digestibility on AA and low energy content by high fiber content can be an obstacle for using CM. This experiment was conducted to evaluate the effects of CM supplementation with *β*-mannanase on growth performance, blood profile, nutrient digestibility, pork quality and economic analysis in growing-finishing pigs.

**Methods:**

A total of 100 growing pigs ([Yorkshire × Landrace] × Duroc) averaging 31.22 ± 2.04 kg body weight were allotted to 5 different treatments by weight and sex in a randomized complete block (RCB) design in 5 replicate with 4 pigs per pen. Treatments were 1) Control (corn-SBM based diet + 0.1% of *β*-mannanase (800 IU)), 2) CM10 (10% copra meal + 0.1% *β*-mannanase (800 IU)), 3) CM15 (15% copra meal + 0.1% *β*-mannanase (800 IU)), 4) CM20 (20% copra meal + 0.1% *β*-mannanase (800 IU)) and 5) CM25 (25% copra meal + 0.1% *β*-mannanase (800 IU)). Four phase feeding program was used: growing I (week 1–3), growing II (week 4–6), finishing I (week 7–9) and finishing II (week 10–12).

**Results:**

In growth performance, there was no significant difference among treatments during whole experimental period. In growingI phase, G:F ratio tended to increase when CM was increased (P = 0.05), but ADG and ADFI tended to decrease in finishingII phase (linear, *P* = 0.08). Also, increasing CM reduced ADG (linear, *P* = 0.02) and feed efficiency (linear, *P* = 0.08) during the whole finishing period. In blood profiles, BUN was linearly increased as CM increased (linear, *P* = 0.02) at growingII period. In digestibility trial, there was no significant difference in dry matter, crude fat, crude ash and nitrogen digestibility. However, crude protein digestibility was decreased linearly (linear, *P* = 0.02). In economic analysis, feed cost per weight gain and total feed cost per pig were reduced in overall period when CM was provided by 25% (linear, *P* = 0.02).

**Conclusion:**

CM with 0.1% of *β*-mannanase (800 IU) could be supplemented instead of corn and SBM up to 25% without detrimental effects on growth performance and pork quality of growing-finishing pigs.

## Background

Corn, SBM and wheat occupy the highest part of feed cost. To reduce the amount of those ingredients which were generally used in swine diet, various alternative ingredients such as palm kernel meal (PKM), CM, sorghum and others have been tested [1–3]. Those are needed to be an alternative feed ingredients which are sufficient production, stable supply, storage convenience and cheaper price than established ingredients [4]. CM fits to those required conditions and its production is currently emerging. There are more advantages in the amount of production and low price compared to corn or SBM as the by-product of oil extraction from coconut. CM has been extensively used in tropical regions that production amount is high enough to provide energy [5] or protein [6]. However, CM has disadvantages of low digestibility on essential amino acids [2] and low energy content [7] because of high dietary fiber (48.8%, in DM; dry matter) [8].

These fiber sources in CM are mostly non-soluble dietary fiber (33.6%, in DM) [8] and its main form is generally known mannose [9]. The mannose in CM has *β*-1,4-mannose chain structure and α-1,6-galactose side chain [10, 11].

The great part of non-starch polysaccharides (NSP) in CM is mannan, and its proportion is 25 ~ 30% on a DM basis [9]. It suggests that the availability of the mannan can be improved with mannanase supplementation and consequently the range of usage for feed can be extended. Lee [12] demonstrated that 400 IU of *β*-mannanase supplementation to growing-finishing pigs tended to show the better growth performance, intestinal flora and nutrient digestibility in corn-SBM based diets. 800 IU level of *β*-mannanase was calculated by more amount of mannan substrate in CM. Based on these background, this experiment was conducted to evaluate effects of various level of CM with *β*-mannanase supplementation on growth performance, blood profile, nutrient digestibility, pork quality and economic analysis in growing-finishing pigs.

## Methods

All experimental procedures involving animals were conducted in accordance with the Animal Experimental Guidelines provided by the Seoul National University Institutional Animal Care and Use Committee (SNUIACUC; SNU-160613-10).

### Animals and feeding trial

A total of 100 crossbred pigs ([Yorkshire × Landrace] × Duroc) with an average body weight of 31.02 ± 2.04 kg were used for 12 week feeding trial in experimental farm of Seoul National University. Pigs were grouped by body weight and sex, and assigned to five treatments according to RCB design. Each treatment had 5 replicates with 4 pigs per pen. Pigs were housed in growing pen (1.40 × 2.50 m^2^) and finishing pen (1.70 × 2.50 m^2^) that were easy to supply feed and water by *ad libitum* and control room temperature and ventilation. Body weight and feed intake were recorded at every 3 weeks to calculate the average daily gain (ADG), average daily feed intake (ADFI) and gain to feed ratio (G:F ratio) of the pigs.

### Experimental design and feeding program

The treatments included 1) Control: 5% of copra meal + 0.1% of β-mannanase (800 IU), 2) CM10: 10% of copra meal + 0.1% of β-mannanase (800 IU), 3) CM15: 15% of copra meal + 0.1% of β-mannanase (800 IU), 4) CM20: 20% of copra meal + 0.1% of β-mannanase (800 IU), 5) CM25: 25% of copra meal + 0.1% of *β*-mannanase (800 IU). Mainly corn, SBM and wheat bran were replaced by the CM. The diets were formulated to contain 3,265 kcal of ME/kg for the all phases. Experimental diets were mixed mainly with corn and SBM and nutrients of experimental diets were met or exceeded the requirement of NRC [13]. *β*-mannanase (patent, 10-0477456-0000; CTCbio® Inc., Seoul, Republic of Korea) in dry form was supplemented in basal diet.

The feeding program was composed with four phases; growing I (week 1–3), growing II (week 4–6), finishing I (week 7–9) and finishing II (week 10–12) respectively. Formula and chemical composition of diet were presented in Tables 1, 2, 3 and 4. Expeller CM from Philippines was used in this experiment, and the analyzed composition was shown in Table 5.

**Table 1:** Formula and chemical composition of the diet in growing phase I (0–3 week)

Criteria	Treatment^a^				
	Con	CM10	CM15	CM20	CM25
Ingredient, %					
Corn	54.65	53.23	51.82	50.37	48.94
SBM-45	25.07	23.41	21.79	20.09	18.47
Wheat bran	9.88	7.96	5.99	4.17	2.23
Copra meal	5.00	10.00	15.00	20.00	25.00
Soy oil	2.76	2.76	2.76	2.76	2.76
DCP	1.17	1.09	1.02	0.90	0.90
Limestone	0.73	0.77	0.81	0.86	0.90
L-lysine HCl	0.14	0.18	0.21	0.25	0.28
Vit. Mix^b^	0.10	0.10	0.10	0.10	0.10
Min. Mix^c^	0.10	0.10	0.10	0.10	0.10
Salt	0.30	0.30	0.30	0.30	0.30
*β*-mannanase^d^	0.10	0.10	0.10	0.10	0.10
Total	100.00	100.00	100.00	100.00	100.00
Chemical composition^e^					
ME, kcal/kg	3,265.00	3,265.01	3,265.01	3,265.00	3,265.00
CP, %	18.00	18.00	18.00	18.00	18.00
Lysine, %	0.95	0.95	0.95	0.95	0.95
Methionine, %	0.26	0.26	0.26	0.27	0.27
Ca, %	0.60	0.60	0.60	0.60	0.60
Total P, %	0.50	0.50	0.50	0.50	0.50

^a^Con : 5% copra meal + 0.1% *β*-mannanase, C10 : 10% copra meal + 0.1% *β*-mannanase, C15 : 15% copra meal + 0.1% *β*-mannanase, C20 : 20% copra meal + 0.1% *β*-mannanase, C25 : 25% copra meal + 0.1% *β*-mannanase

^b^Provided per kg of diet: Vit A, 16,000 IU; Vit D_3_, 3,200 IU; Vit E, 35 IU; Vit K3, 5 mg; Rivoflavin, 6 mg; Cacium pantothenic acid, 16 mg; Niacin, 32 mg; d-Biotin, 128 μg, Vit B_12_, 20 μg

^c^Provided per kg of diet: Fe, 281 mg; Cu, 288 mg; Mn, 49 mg; I, 0.3 mg; Se, 0.3 mg

^d^CTCzyme®: CTCbio Inc. Seoul, Republic of Korea

^e^Calculated value

**Table 2:** Formula and chemical composition of the diet in growing phase II (4–6 week)

Criteria	Treatment^a^				
	Con	CM10	CM15	CM20	CM25
Ingredient, %					
Corn	58.26	56.84	55.39	53.98	52.55
SBM-45	20.35	18.69	16.99	15.37	13.72
Wheat bran	11.21	9.29	7.47	5.50	3.58
Copra meal	5.00	10.00	15.00	20.00	25.00
Soy oil	2.74	2.74	2.74	2.74	2.74
DCP	1.11	1.02	0.92	0.81	0.77
Limestone	0.63	0.68	0.71	0.79	0.79
L-lysine · HCl	0.10	0.14	0.18	0.21	0.25
Vit. Mix^b^	0.10	0.10	0.10	0.10	0.10
Min. Mix^c^	0.10	0.10	0.10	0.10	0.10
Salt	0.30	0.30	0.30	0.30	0.30
*β*-mannanase^d^	0.10	0.10	0.10	0.10	0.10
Total	100.00	100.00	100.00	100.00	100.00
Chemical composition^e^					
ME, kcal/kg	3,265.00	3,265.01	3,265.01	3,265.00	3,265.00
CP, %	16.30	16.30	16.30	16.30	16.30
Lysine, %	0.82	0.82	0.82	0.82	0.82
Methionine, %	0.25	0.25	0.25	0.25	0.25
Ca, %	0.54	0.54	0.54	0.54	0.54
Total P, %	0.47	0.47	0.47	0.47	0.47

^a^Con : 5% copra meal + 0.1% *β*-mannanase, C10 : 10% copra meal + 0.1% *β*-mannanase, C15 : 15% copra meal + 0.1% *β*-mannanase, C20 : 20% copra meal + 0.1% *β*-mannanase, C25 : 25% copra meal + 0.1% *β*-mannanase

^b^Provided per kg of diet: Vit A, 16,000 IU; Vit D_3_, 3,200 IU; Vit E, 35 IU; Vit K3, 5 mg; Rivoflavin, 6 mg; Cacium pantothenic acid, 16 mg; Niacin, 32 mg; d-Biotin, 128 μg, Vit B_12_, 20 μg

^c^Provided per kg of diet: Fe, 281 mg; Cu, 288 mg; Mn, 49 mg; I, 0.3 mg; Se, 0.3 mg

^d^CTCzyme®: CTCbio Inc. Seoul, Republic of Korea

^e^Calculated value

**Table 3:** Formula and chemical composition of the diet in finishing phase I (7–9 week)

Criteria	Treatment^a^				
	Con	CM10	CM15	CM20	CM25
Ingredient, %					
Corn	59.93	58.50	57.06	55.67	54.24
SBM-45	18.10	16.43	14.77	13.15	11.50
Wheat bran	11.96	10.09	8.20	6.20	4.28
Copra meal	5.00	10.00	15.00	20.00	25.00
Soy oil	2.72	2.72	2.72	2.72	2.72
DCP	1.03	0.94	0.83	0.77	0.69
Limestone	0.58	0.61	0.67	0.71	0.75
L-lysine HCl	0.08	0.11	0.15	0.18	0.22
Vit. Mix^b^	0.10	0.10	0.10	0.10	0.10
Min. Mix^c^	0.10	0.10	0.10	0.10	0.10
Salt	0.30	0.30	0.30	0.30	0.30
*β*-mannanase^d^	0.10	0.10	0.10	0.10	0.10
Total	100.00	100.00	100.00	100.00	100.00
Chemical composition^e^					
ME, kcal/kg	3,265.00	3,265.00	3,265.01	3,265.01	3,265.00
CP, %	15.50	15.50	15.50	15.50	15.50
Lysine, %	0.75	0.75	0.75	0.75	0.75
Methionine, %	0.24	0.24	0.24	0.25	0.25
Ca, %	0.50	0.50	0.50	0.50	0.50
Total P, %	0.45	0.45	0.45	0.45	0.45

^a^Con : 5% copra meal + 0.1% *β*-mannanase, C10 : 10% copra meal + 0.1% *β*-mannanase, C15 : 15% copra meal + 0.1% *β*-mannanase, C20 : 20% copra meal + 0.1% *β*-mannanase, C25 : 25% copra meal + 0.1% *β*-mannanase

^b^Provided per kg of diet: Vit A, 16,000 IU; Vit D_3_, 3,200 IU; Vit E, 35 IU; Vit K3, 5 mg; Rivoflavin, 6 mg; Cacium pantothenic acid, 16 mg; Niacin, 32 mg; d-Biotin, 128 μg, Vit B_12_, 20 μg

^c^Provided per kg of diet: Fe, 281 mg; Cu, 288 mg; Mn, 49 mg; I, 0.3 mg; Se, 0.3 mg

^d^CTCzyme®: CTCbio Inc. Seoul, Republic of Korea

^e^Calculated value

**Table 4:** Formula and chemical composition of the diet in finishing phase II (10–12 week)

Criteria	Treatment^a^				
	Con	CM10	CM15	CM20	CM25
Ingredient, %					
Corn	64.84	63.42	62.00	60.58	59.15
SBM-45	11.65	9.99	8.33	6.67	5.02
Wheat bran	13.71	11.79	9.87	7.97	6.05
Copra meal	5.00	10.00	15.00	20.00	25.00
Soy oil	2.70	2.70	2.70	2.70	2.70
DCP	0.85	0.78	0.70	0.61	0.52
Limestone	0.59	0.62	0.66	0.70	0.75
L-lysine HCl	0.06	0.10	0.14	0.17	0.21
Vit. Mix^b^	0.10	0.10	0.10	0.10	0.10
Min. Mix^c^	0.10	0.10	0.10	0.10	0.10
Salt	0.30	0.30	0.30	0.30	0.30
*β*-mannanase^d^	0.10	0.10	0.10	0.10	0.10
Total	100.00	100.00	100.00	100.00	100.00
Chemical composition^e^					
ME, kcal/kg	3,265.00	3,265.02	3,265.04	3,265.01	3,265.00
CP, %	13.20	13.20	13.20	13.20	13.20
Lysine, %	0.60	0.60	0.60	0.60	0.60
Methionine, %	0.22	0.22	0.22	0.23	0.23
Ca, %	0.45	0.45	0.45	0.45	0.45
Total P, %	0.40	0.40	0.40	0.40	0.40

^a^Con: 5% copra meal + 0.1% *β*-mannanase, C10 : 10% copra meal + 0.1% *β*-mannanase, C15 : 15% copra meal + 0.1% *β*-mannanase, C20 : 20% copra meal + 0.1% *β*-mannanase, C25 : 25% copra meal + 0.1% *β*-mannanase

^b^Provided per kg of diet: Vit A, 16,000 IU; Vit D_3_, 3,200 IU; Vit E, 35 IU; Vit K3, 5 mg; Rivoflavin, 6 mg; Cacium pantothenic acid, 16 mg; Niacin, 32 mg; d-Biotin, 128 μg, Vit B_12_, 20 μg

^c^Provided per kg of diet: Fe, 281 mg; Cu, 288 mg; Mn, 49 mg; I, 0.3 mg; Se, 0.3 mg

^d^CTCzyme®: CTCbio Inc. Seoul, Republic of Korea

^e^Calculated value

**Table 5:** Analyzed composition of CM for experiment

Analyzed composition, %	
Crude protein	19.50
Ether extract	07.35
Moisture	09.09
Crude Ash	05.94

### Blood profiles

Blood samples were collected from anterior vena cava of 6 pigs per each treatment at 0, 3, 6, 9 and 12 week for analyzing concentration of glucose and blood urea nitrogen (BUN) in serum. Collected blood samples were centrifuged for 15 min at 3,000 rpm in 4 °C (Eppendorf centrifuge 5810R, Germany). Serum was carefully removed to microtubes and stored at −20 °C until analysis. Glucose and BUN concentration were analyzed using a blood analyzer (Ciba-Corning model, Express Plus, Ciba Corning Diagnostics Co.).

### Nutrient digestibility

A total of 15 crossbred barrows, average body weight 34.73 ± 0.24 kg, were allocated to the each five treatment with three replicates in metabolic cages in a completely randomized design (CRD). The experimental diets were provided twice daily by 2.0% of body weight (on 1% of in each feeding) at 7:00 and 19:00, and water was provided by ad libitum. After 5 days of adaptation period, feces and urine were collected for further 5 days by the method of Hong et al. [14]. The feces and urine collected from each pig during these 5 days were stored at −20 °C until they were analyzed. After collecting period, the excreta were dried in an air-forced drying oven at 60 °C for 72 h and ground to 2 mm of diameter by a Wiley mill for chemical analysis [15]. Feed, feces and urine were chemically analyzed by the method of AOAC [15].

### Carcass traits

At the end of experiment, four pigs from each treatment group were selected and slaughtered at average 117.8 ± 1.06 kg for the carcass analysis. Pork samples were collected from nearby 10th rib on right side of carcass. Because of chilling procedure, 30 min after slaughter was regarded as initial time. The pH and pork color were measured at 0, 3, 6, 9, 12 and 24 h, respectively. The pH was measured using a pH meter (Bechman Coulter Φ 500 Series, USA) and pork color was determined by CIE color L*, a*, and b* values using a CR300 (Minolta Camera Co., Japan). Chemical analysis of pork samples were conducted by the method of AOAC [15].

### Pork quality

Water holding capacity of pork was measured by centrifuge method (Ryoichi et al., 1993). Longissimus muscles were ground and sampled in filter tube, then heated in water bath at 80 °C for 20 min and centrifuged for 10 min at 2,000 rpm and 4 °C (Eppendorf centrifuge 5810R, Germany). After that, to calculate the cooking loss, longissimus muscles were packed with polyethylene bag and heated in water bath until core temperature reached 70 °C and weighed before and after cooking. After heated, samples were cored (0.5 in. in diameter) parallel to muscle fiber and the cores were used to measure the shear force using a salter (Warner Bratzler Shear, USA). Shear force, cooking loss and water holding capacity of pork were analyzed by National Institute of Animal Science.

### Economic analysis

As the experimental pigs were reared in the same environmental condition, economical efficiency was calculated by considering only the feed cost. The feed cost per body weight gain (won/kg) was calculated using total feed intake and feed price. The days to reaching market weight (115 kg) was estimated from the body weight at the end of feeding trial and ADG of 9–12 week.

### Statistical analysis

All data were analyzed using the general linear model (GLM) procedure of SAS [16]. RCB design was adopted in feeding trial and pen was the experimental block unit. CRD was adopted in digestibility trial, blood and carcass analysis and individual pig was the experimental unit. Statistical differences were considered significant at the level of *P* < 0.05 and highly significant at the level of *P* < 0.01, with a trend between *P* ≥ 0.05 and *P* ≤ 0.10 for Control group and CM treated group. Statistical differences were considered to be linear or quadratic at the level of *P* < 0.05 and highly linear or quadratic at the level of *P* < 0.01 for CM treated group.

## Results and Discussion

### Growth performance

The effect of CM supplementation with β-mannanase on growth performance during 12 weeks was presented in Table 6. In growing phase, the level of CM did not affect the body weight (BW), ADG and ADFI. However, G:F ratio tended to increase when CM level was increased in growing I phase (linear, *P* = 0.05). In finishing I phase, tendency of quadratic response was observed on CM20 treatment (quadratic, *P* = 0.06). During finishing II period, the ADG and ADFI tended to decrease when CM level increased (linear, *P* = 0.08, P = 0.06, respectively). Also, a linear response on the ADG (linear, *P* = 0.02) and tendency of decreasing in G:F ratio (linear, *P* = 0.08) were observed as increasing level of CM in diet during the whole finishing period.

**Table 6:** Effect of dietary levels of copra meal with β-mannanase on growth performance in growing-finishing pigs^a^

Criteria	Treatment					SEM^b^	*P*-value	
	Con	CM10	CM15	CM20	CM25		Lin.	Quad.
Body weight, kg								
Initial	31.07	30.98	30.98	31.07	30.88	0.543	–	–
3 week	41.32	41.34	42.05	43.77	42.12	0.740	0.25	0.56
6 week	60.40	59.08	60.47	63.96	61.73	1.143	0.20	0.97
9 week	82.16	82.17	83.22	85.94	83.17	1.264	0.33	0.57
12 week	101.77	101.93	99.95	101.98	99.91	1.172	0.61	0.96
ADG, g								
0–3 week	488	493	532	606	524	19.9	0.25	0.46
4–6 week	909	845	877	962	934	25.8	0.34	0.54
7–9 week	1,036	1,099	1,083	1,047	1,020	16.9	0.49	0.19
10–12 week	934	941	797	764	797	36.1	0.08	0.56
0–6 week	698	669	704	784	729	19.0	0.19	0.96
7–12 week	985	1,020	940	905	909	17.1	0.02	0.87
0–12 week	842	844	822	845	819	9.8	0.57	0.89
ADFI, g								
0–3 week	1,557	1,464	1,415	1,596	1,406	47.0	0.63	0.93
4–6 week	2,143	2,051	2,124	2,129	2,177	56.8	0.73	0.66
7–9 week	2,969	3,024	2,894	3,037	3,291	62.4	0.12	0.17
10–12 week	3,077	3,060	2,782	2,863	2,602	74.4	0.06	0.84
0–6 week	1,850	1,758	1,769	1,862	1,791	45.8	0.97	0.75
7–12 week	3,023	3,042	2,838	2,950	2,947	49.0	0.55	0.57
0–12 week	2,437	2,400	2,303	2,406	2,369	43.3	0.70	0.62
G:F ratio								
0–3 week	0.310	0.340	0.378	0.383	0.373	0.0113	0.05	0.24
4–6 week	0.423	0.411	0.412	0.461	0.433	0.0119	0.51	0.88
7–9 week	0.350	0.363	0.376	0.346	0.311	0.0082	0.11	0.05
10–12 week	0.304	0.310	0.282	0.269	0.308	0.0108	0.67	0.40
0–6 week	0.376	0.381	0.398	0.427	0.408	0.0083	0.11	0.63
7–12 week	0.327	0.336	0.331	0.308	0.309	0.0057	0.08	0.39
0–12 week	0.346	0.353	0.357	0.353	0.346	0.0038	0.97	0.28

^a^A total of 100 crossbred pigs was fed from average initial body weight 31.02 ± 2.04 kg to average final body weight 100.17 kg

^b^Standard error of the mean

Nunes and Malmlof [17] reported that high level of mannan in swine diet interfered with the insulin and IGF-I secretion and affected negatively on growth performance. But, in current experiment, CM25 showed similar growth performance to CM5 without statistical difference in growing period and the whole experimental period. This can be explained by result of Khanongnuch et al. [18] that exogenous mannanase which degraded mannan in CM was sufficient even in CM25, so that they had enough transit time to digest ingesta including CM. Petty et al. [19] also reported adding β-mannanase to diets had a positive influence on pigs and improved the G:F ratio (*P* < 0.01).

Similarly, supplementation of mannan degrading enzyme to growing-finishing pig diet has increased G:F ratio and lean gain [20, 21]. However, in finishing II phase and during whole finishing period, ADFI was decreased in agreement with Jaworski et al. [22] who demonstrated that supplementation of diet with CM lowered feed intake. Also, reduction of ADFI by CM inclusion may affect the ADG and G:F ratio in finishing period [22]. It is considered to be due to rancidity after prolonged storage [23] or the lower density and more swelling (high crude fiber) of CM [18].

This result demonstrated that palatability was affected by level of 20% CM in finishing phase. However, ADFI in growing phase was not affected, and there was an improvement of feed efficiency by increasing level of CM in early growing phase. These observations were supported by the result of former researchers [22, 23].

Consequently, during the whole experimental period, there were no significant differences in growth performance when fed up to 25% of CM with 0.10% of *β*-mannanase.

### Blood profiles

**Fig. 1:**
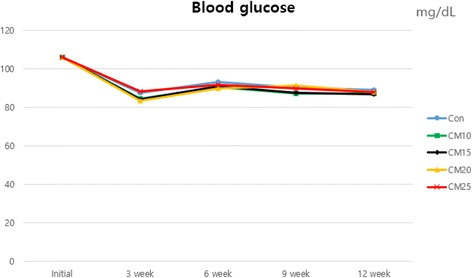
Effect of dietary levels of copra meal with β-mannanase on blood glucose in growing-finishing pigs

**Fig. 2:**
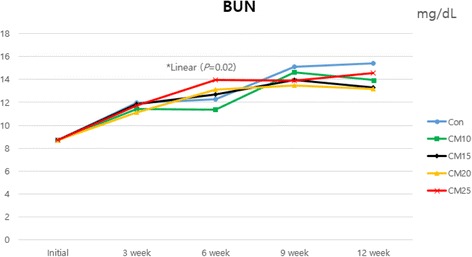
Effect of dietary levels of copra meal with β-mannanase on blood urea nitrogen (BUN) in growing-finishing pig

**Fig. 3:**
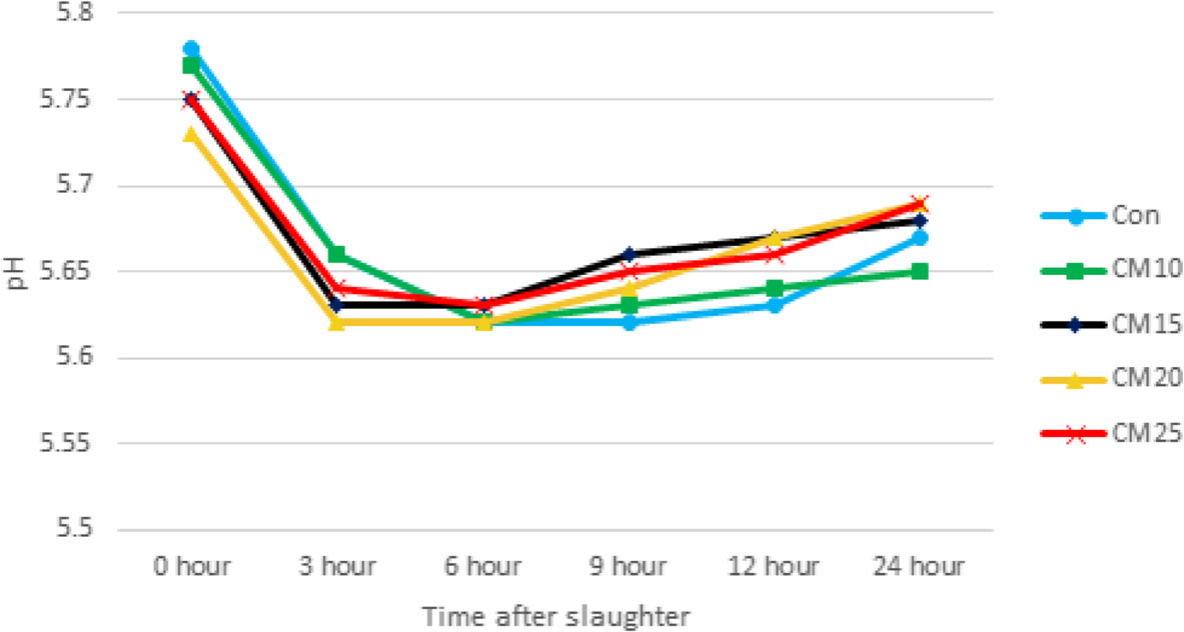
Effect of dietary levels of copra meal with *β*-mannanase on pH at slaughtering

Blood profiles parameters during feeding trial were presented in Figs. 1, 2 and 3. During the whole experimental period, there was no significant difference in glucose concentration when pigs were fed diet with increasing level of CM. Mannans and galactomannans in CM might reduce the absorption of glucose and decrease the production of insulin [17, 24]. But, Kim et al. [25], demonstrated that the concentration of blood glucose in pigs fed diet with *β*-mannanase was greater than pigs fed diet without *β*-mannanase (*P* = 0.03). The result of this study was considered as due to the reduced negative effects of anti-nutritional factor by added dietary *β*-mannanase [26]. At 6 week, linear increasing of BUN was observed as CM supplementation level increased (linear, *P* = 0.02). The BUN has been known to a good indicator for evaluation in protein quality, protein intake [27] and nitrogen retention [28] by pigs. Münchow and Bergner [29] reported that there was a highly negative correlation between the biological value of feed and BUN content. Increase of BUN concentration indicated that excessive amino acids are inefficiently metabolized and circulated in the blood before excretion [30, 31]. Although a linear response by CM supplementation level was observed at 6 week, the BUN value of all treatments were in normal range (10.0 – 30.0 mg/dL). Consequently, CM inclusion with *β*-mannanase did not negatively affect the blood glucose and BUN.

### Nutrient digestibility

The effect of CM supplementation with *β*-mannanase on nutrient digestibility was presented in Table 7. The digestibility of crude protein in growing pigs was linearly declined with increasing inclusion of CM (linear, *P* = 0.02), and the tendency of decrease was found when over 15% of CM was supplemented (*P* = 0.07). The digestibility of dry matter, crude fat, and crude ash showed no significant difference among treatments. Nitrogen retention tended to be decreased (linear, *P* = 0.08) as CM level increased in pig diet, leading to the tendency of increasing in fecal nitrogen (linear, *P* = 0.05).

**Table 7:** Effect of dietary levels of copra meal with β-mannanase on nutrient digestibility in growing pigs^a^

Criteria	Treatment					SEM^b^	*P*-value	
	Con	CM10	CM15	CM20	CM25		Lin.	Quad.
Nutrient digestibility (%)								
Dry matter	85.69	86.79	83.65	84.60	85.82	0.433	0.39	0.12
Crude protein	83.19	83.93	80.95	80.76	80.68	0.551	0.02	0.73
Crude fat	56.71	58.13	56.35	55.82	58.32	0.976	0.49	0.63
Crude ash	65.47	67.57	60.99	64.45	64.00	1.184	0.87	0.60
Nitrogen retention^c^, g/day								
N-intake	16.60	16.74	16.70	16.58	16.49	0.186	0.75	0.71
N-feces	2.80	2.69	3.19	3.20	3.19	0.112	0.05	0.66
N-urine	10.17	10.55	9.95	10.21	10.22	0.141	0.84	0.94
N-retention	3.63	3.50	3.55	3.16	3.09	0.127	0.08	0.70
N-digestibility (%)	21.81	20.90	21.24	19.10	18.73	0.692	0.13	0.81

^a^A total of 15 barrows with an initial body weight 34.7 ± 0.24 kg

^b^Standard error of the mean

^c^N retention = N intake – Fecal N – Urinary N

During heating process of CM, the Maillard reaction occurred between mannose and amino group which alleviated total nutritional value of CM [32]. Also, Kim et al. [3] demonstrated that lowered digestibility was observed when pigs were fed CM because of the higher crude fiber level in CM than in SBM. The negative effect of fiber might be from the effect of fiber on transit time, water-binding capacity of fiber, mechanical erosion and absorption of nutrients on the fiber [33].

Consequently, increasing level of CM caused lower crude protein (CP) digestibility by higher fiber which was the reason of increased fecal nitrogen and reduced nitrogen retention.

### Carcass traits and pork quality

The effect of CM supplementation with *β*-mannanase on pH of pork was presented in Fig. 3. In this study, increasing level of CM did not affect the pH levels change and the pH value at 0, 24 h.

The pH change of pork is a very important factor that determines the quality of pork and has an influence on freshness, tenderness, meat color and texture [34].

It is also an important factor for the storage [35]. In fact, the initial pH is regarded as an indication of PSE (pale, soft and exudative) pork and the final pH is acknowledged as an estimation of DFD (dark, firm and dry). In general, pH decline is accelerated as time goes by, and ultimate pH is reduced because of creation of lactic acid from glycogen [36–38]. In this study, since the CM level in diet did not affect pH of pork, it is considered that inclusion of CM did not have adverse effect on pork quality which was correlated with pH.

The results of pork color were presented in Table 8. There was no significant difference among treatments in L*, a* and b* value at 0, 3, 6, 9, 12, 24 h after slaughter. In pork color, decreasing in redness and increasing in yellowness had a negative influence on the freshness of pork [39]. But, there was no change in redness or yellowness by CM level in diet. These findings were in accordance with those of Hong [40], who had demonstrated that a* and b* values were not affected by CM inclusion level when 0.10% of *β*-mannanase supplemented.

**Table 8:** Effect of dietary levels of copra meal with *β*-mannanase on pork color after slaughter

Criteria	Treatment					SEM^a^	*P-*value	
	Con	CM10	CM15	CM20	CM25		Lin.	Quad.
CIE value^b^, L*								
0 h	44.01	44.60	43.32	42.53	44.32	0.658	0.78	0.64
3 h	44.59	43.33	43.88	43.28	44.85	0.714	0.93	0.47
6 h	46.30	47.57	46.63	45.13	47.71	0.539	0.92	0.64
9 h	47.61	47.32	47.53	46.50	48.86	0.482	0.50	0.19
12 h	47.59	47.00	46.70	46.52	48.24	0.517	0.80	0.23
24 h	48.37	46.49	48.17	47.72	49.30	0.593	0.34	0.22
CIE value, a*								
0 h	2.47	2.85	2.35	2.00	2.32	0.128	0.18	0.98
3 h	3.19	2.92	2.43	2.51	2.78	0.113	0.15	0.11
6 h	3.17	3.81	2.67	2.48	2.81	0.189	0.12	0.82
9 h	3.52	3.35	3.04	2.91	3.69	0.153	0.93	0.15
12 h	3.81	4.08	3.30	3.03	4.00	0.156	0.52	0.13
24 h	4.37	4.12	3.97	3.86	4.40	0.132	0.81	0.13
CIE value, b*								
0 h	4.88	4.79	4.68	4.27	4.54	0.130	0.22	0.70
3 h	5.57	4.79	4.92	4.82	5.05	0.151	0.33	0.15
6 h	5.68	5.69	5.36	5.01	5.50	0.156	0.35	0.48
9 h	5.93	5.64	5.61	5.57	6.19	0.143	0.63	0.12
12 h	6.07	5.98	5.75	5.76	6.19	0.136	0.96	0.22
24 h	6.72	6.29	6.27	6.29	6.56	0.135	0.68	0.11

^a^Standard error of the mean

^b^CIE L: luminance or brightness (vary from black to white), a: red · green component (+a = red, −a = green), b: yellow · blue component (+b = yellow, −b = blue)

The effects of on the carcass characteristics of growing-finishing pigs fed with increasing level of CM with *β*-mannanase were noted in Table 9. In current study, the level of CM did not affect the cooking loss, shear force, water holding capacity (WHC) and proximate analysis of the pork after slaughter. The result of fat content contrasted with those of Creswell and Brooks [2] who demonstrated that the relationship between dietary CM and fat composition of carcass and CM additions resulted in increased fatty acids in the backfat. When crude fat content increases in longissimus muscles, WHC is increased and shear force and cooking loss are decreased [41].

**Table 9:** Effect of dietary levels of copra meal with *β*-mannanase on pork quality

Criteria	Treatment					SEM^a^	*P*-value	
	Con	CM10	CM15	CM20	CM25		Lin.	Quad.
Proximate analysis, %								
Moisture	66.10	65.52	65.34	66.01	65.26	0.142	0.23	0.65
Crude protein	22.62	22.82	22.22	23.11	22.97	0.150	0.31	0.48
Crude fat	1.72	1.76	1.69	1.61	1.62	0.093	0.64	0.93
Crude ash	9.94	9.97	9.94	10.14	10.15	0.082	0.41	0.80
Physiochemical property								
Cooking loss, %	32.07	30.99	33.13	31.34	33.18	0.334	0.24	0.45
Shear force^b^	3.32	3.27	3.40	3.01	3.36	0.086	0.78	0.73
WHC, %	56.74	55.67	55.01	55.14	54.93	0.322	0.11	0.39

^a^Standard error of the mean

^b^kg/0.5 in

### Economic analysis

The effects of dietary CM levels with 0.10% of β-mannanase on feed cost were presented in Table 10. There was a tendency in feed cost per weight gain in growing I phase (linear, *P* = 0.06), growing II phase (linear, *P* = 0.09). During overall period, there was decreasing in (linear, *P* = 0.02) feed cost per weight gain according to effect of dietary CM levels increasing, either. Also, total feed cost per pig was reduced by dietary CM levels in growing I phase (linear, *P* = 0.03) and during overall period (linear, *P* = 0.04). Decreasing feed cost of high content of CM was mainly caused by the replacement of SBM. There was no significant response in days to market weight. Total feed cost in whole days to market weight was reduced up to 6.56% when fed diets with increasing level of CM. Also, feed cost (won/kg) reduction of 1.01 won was observed when each 1% CM inclusion level increased.

**Table 10:** Effect of dietary levels of copra meal with *β*-mannanase on economic analysis

Criteria	Treatment^a^					SEM^a^	*P*-value	
	Con	CM10	CM15	CM20	CM25		Lin.	Quad.
Feed cost per weight gain, won/kg								
0–3 week	1,230	1,386	1,142	1,063	1,041	51.7	0.06	0.64
4–6 week	954	1,028	977	875	900	21.0	0.09	0.40
7–9 week	1,123	1,185	1,110	1,136	1,184	17.1	0.56	0.63
10–12 week	1,246	1,081	1,211	1,260	1,122	27.4	0.61	0.89
Total	4,553	4,679	4,440	4,334	4,245	58.8	0.02	0.49
Total feed cost per pig, won/head								
0–3 week	13,379	12,388	11,977	11,998	11,292	293.5	0.03	0.64
4–6 week	17,646	17,059	17,289	17,228	16,570	374.1	0.45	0.89
7–9 week	24,517	25,029	23,845	23,594	24,041	300.5	0.29	0.76
10–12 week	42,491	42,736	41,083	41,222	39,700	1,688.5	0.48	0.89
Total	98,033	97,212	94,194	94,042	91,603	1,245.8	0.04	0.95
Relative ratio to control	100.00	99.16	96.08	95.93	93.44	–	–	–
Days to market weight (reached at 115 kg BW)								
	101.1	101.7	101.9	104.0	104.0	1.50	0.46	0.95

^a^Standard error of the mean

## Conclusion

In conclusion, there is a possibility for CM as an alternative feed ingredient in growing to finishing pigs up to 25%. In growth performance, there was no detrimental effects in 25% of CM treatment with 0.10% of *β*-mannanase. Concentration of glucose and BUN showed general levels but linear increase of BUN was found as CM level increased. Pork quality was not affected by CM supplementation level. Although nutritional digestibility was linearly decreased as CM content increased, economic efficiency was linearly improved during the whole experimental period. Consequently, CM as an alternative feed stuff could be supplemented in growing to finishing pig diets up to 25% when *β*-mannanase was supplemented.

## Abbreviations

ADFI: Average daily feed intake
ADG: Average daily gain
BUN: Blood urea nitrogen
BW: Body weight
CM: Copra meal
CP: Crude protein
CRD: Completely randomized design
DM: Dry matter
G:F ratio: Gain to feed ratio
GLM: General linear model
NSP: Non-starch polysaccharide
PKM: Palm kernel meal
RCB: Randomized complete block
SBM: Soy bean meal
WHC: Water holding capacity

## References

[1] Agunbide JA, Wiseman J, Cole DJA (1999). Energy and nutrient use of palm kernels, palm kernel meal and palm kernel oil in diets for growing pigs. Anim Feed Sci Technol.

[2] Creswell DC, Brooks CC (1971). Compositions, apparent digestibility and energy evaluation of coconut oil and coconut meal. J Anim Sci.

[3] Kim BG, Lee HH, Jung HJ, Han YK, Park KM, Han IK (2001). Effect of partial replacement of soybean meal with palm kernel meal and copra meal on growth performance, nutritional digestibility and carcass characteristics of finishing pigs. Asian Aust J Anim Sci.

[4] Biavatti MW, Bellaver MH, Volpato L, Costa C, Bellaver C (2003). Preliminary studies of alternative feed additives for broilers: Alternanthera brasiliana extract, propolis extract and linseed oil. J Poult Sci.

[5] O’ Doherty JV, McKeon MP (2000). The use of expeller copra meal in grower and finisher pig diets. Livest Prod Sci.

[6] Dung NNX, Manh LH, Uden P (2002). Tropical fiber sources for pigs–digestibility, digesta retention and estimation of fibre digestibility in vitro. Anim Feed Sci Technol.

[7] Sauvant D, Perez JM, Tran G (2004). Tables of composition and nutritional value of feed materials.

[8] Knudsen KEB (1997). Carbohydrate and lignin contents of plant materials used in animal feeding. Anim Feed Sci Technol.

[9] Saittagaroon S, Kawakishi S, Namiki M (1983). Characterrization of polysaccharides of copra meal. J Sci Food Agri.

[10] Dhawan S, Kaur J (2007). Microbial mannanases: an overview of production and application. Crit Rev Biotechnol.

[11] Hossain MZ, Abe J, Hizukuri S (1996). Multiple forms of *β*-mannanase from *Bacillus sp*. KK01. Enzyme Microb Technol.

[12] Lee JH (2006). Effects of mannose on growth performance, intestinal flora and nutrient digestibility in growing-finishing pigs.

[13] NRC (1998). Nutrient requirements of swine.

[14] Hong JS, Lee GI, Jin XH, Kim YY (2016). Effect of dietary energy levels and phase feeding by protein levels on growth performance, blood profiles and carcass characteristics in growing-finishing pigs. J Anim Sci Technol.

[15] AOAC (1995). Official Methods of Analysis.

[16] Statistical Analysis Systems Institute (2013). SAS user’guide: statistics. version 9.4.

[17] Nunes CS, Malmlof K (1992). Effects of guar gum and cellulose on glucose absorption, hormonal release, and hepatic metabolism in the pig. Brit J Nutr.

[18] Khanongnuch C, Sa-nguansook C, Lumyong S (2006). Nutritive quality of mannanase treated copra meal in broiler diets and effectiveness on some fecal bacteria. Int J Poult Sci.

[19] Petty LA, Carter SD, Senne BW, Shriver JA (2002). Effects of beta-mannanase addition to corn-soybean meal diets on growth performance, carcass traits, and nutrient digestibility of weaning and growing-finishing pig. J Anim Sci.

[20] Hahn JD, Gahl MJ, Giesemann MA, Holsgraefe DP, Fodge DW (1995). Diet type and feed form effects on the performance of finishing swine fed the *β*-mannanase enzyme product Hemicell®. J Anim Sci.

[21] Jo JK, Ingale SL, Kim JS, Kim YW, Kim KH, Lohakare JD, Lee JH, Chae BJ (2012). Effects of exogenous enzyme supplementation to corn and soybean meal-based or complex diets on growth performance, nutrient digestibility, and blood metabolites in growing pigs. J Anim Sci.

[22] Jaworski NW, Shoulders J, Gonzalez-Vega JC, Stein HH (2014). Effects of using copra meal, palm kernel meal in diets in diets in diets for weanling pigs. Prof Anim Sci.

[23] Ehrlich WK, Upton PC, Cowan RT, Moss RJ (1990). Copra meal as a supplement for grazing dairy cows. Proc Aust Soc Anim Prod.

[24] Rainbird AL, Low AG, Zebrowska T (1984). Effect of guar gum on glucose and water absorption from isolated loops of jejunum in conscious growing pigs. Brit J Nutr.

[25] Kim JS, Ingale SL, Lee SH, Kim KH, Kim JS, Lee JH, Chae BJ (2013). Effects of energy levels of diet and *β*-mannananse supplementation on growth performance, apparent total tract digestibility and blood metabolites in growing pigs. Anim Feed Sci Technol.

[26] Yoon SY, Yang YX, Shinde PL, Choi JY, Kim JS, Kim YW, Yun K, Jo JK, Lee JH, Ohh SJ, Kwon IK, Chae BJ (2010). Effects of mannanase and distillers dried grain with soluble on growth performance, nutrient digestibility, and carcass characteristics of grower-finisher pigs. J Anim Sci.

[27] Eggum BO (1970). Blood urea measurement as a technique for assessing protein quality. Brit J Nutr.

[28] Whang KY, Easter RA (2000). Blood urea nitrogen as an index of feed efficiency and lean growth potential in growing-finishing swine. Asian Aust J Anim Sci.

[29] Münchow H, Bergner H (1968). Examination of techniques for protein evaluation of feedstuffs. Archiv Fur Tierenahrung.

[30] Han IK, Lee JH, Piao XS, Defa L (2001). Feeding and management system to reduce environmental pollution in swine production - review. Asian Aust J Anim Sci.

[31] Jeong TS, Heo PS, Lee GY, Kim DH, Ju WS, Kim YY (2001). The influence of phase feeding methods on growth performance, meat quality, and production cost in growing-finishing pigs. J Anim Sci Technol.

[32] Butterworth MH, Fox AC (1963). The effect of heat treatment on the nutritice value of coconut meal and the prediction of the nutritive value by chemical methods. Brit J Nutr.

[33] Nongyao A, Han IK, Choi YJ, Lee NH (1990). Amino acid digestibility as affected by various fiber sources and levels. 1. Difference between ileal and fecal digestibility of amino acids. Asian Aust J Anim Sci.

[34] Kauffman RG, Cassens RG, Scherer A, Meeker DL (1992). Variations in pork quality.

[35] Huff-Lonergan EJ, Baas TJ, Malek M, Dekkers J, Prusa KJ, Rothschild MF (2002). Correlations among selected pork quality traits. J Anim Sci.

[36] Bee G, Biolley C, Guex G, Herzog W, Lonergan SM, Huff-Lonergan E (2006). Effects of available dietary carbohydrate and preslaughter treatment on glycolytic potential, protein degradation, and quality traits of pig muscles. J Anim Sci.

[37] Leheska D, Wulf M, Maddock RJ (2002). Effects of fasting and transportation on pork quality development and extent of postmortem metabolism. J Anim Sci.

[38] Tikk K, Tikk M, Karlsson AH, Andersen HJ. The significance of a muscle glycogen reducing diet on porcine meat and fat color. 51st International Congress of Meat Sci and Technol. 2005;15:15–30.

[39] Bendall JR, Wismer‐Pedersen J (1962). Some properties of the fibrillar proteins of normal and watery pork muscle. J Food Sci.

[40] Hong YG (2009). Different levels of copra meal supplementation with mannanase on growth performance, pork quality and nutrient digestibility in growing-finishing pigs.

[41] Goerl KF, Eilert SJ, Mandigo RW, Chen HY, Miller PS (1995). Pork characteristics as affected by two populations of swine and six crude protein levels. J Anim Sci.

